# Relationship Between Testosterone and Movement Patterns in Male Asian Black Bear ( *Ursus thibetanus* )

**DOI:** 10.1002/ece3.72858

**Published:** 2026-01-11

**Authors:** Naoki Takekoshi, Jumpei Tomiyasu, Yojiro Yanagawa, Naoki Ohnishi, Tomoko Naganuma, Seung‐Yun Baek, Miu Itoh, Xin Luo, Tatsuhito Nishiwaki, Chinatsu Kozakai, Shinsuke Koike, Koji Yamazaki

**Affiliations:** ^1^ Tokyo University of Agriculture Tokyo Japan; ^2^ Obihiro University of Agriculture and Veterinary Medicine Obihiro Japan; ^3^ Faculty of Veterinary Medicine Hokkaido University Sapporo Japan; ^4^ Tohoku Research Center Forestry and Forest Products Research Institute Iwate Japan; ^5^ Tokyo University of Agriculture and Technology Tokyo Japan; ^6^ National Agriculture and Food Research Organization Ibaraki Japan

**Keywords:** consorting, fecal steroid metabolite, movement, testosterone, *Ursus thibetanus*

## Abstract

In male mammals, reproduction and reproductive behavior are strongly influenced by testosterone. While some species exhibit roaming behavior to encounter receptive females and show consorting behavior with females, the relationship between sex hormone and those behaviors remains unclear. To clarify testosterone's influence on behavior, we examined fecal testosterone metabolites (FTM) and behaviors in male Asian black bears ( *Ursus thibetanus* ) using collars equipped with GPS transmitters, activity sensors, and animal‐borne cameras. We assessed four behavioral variables (daily movement speed, net movement distance, turning angle, activity level) and mating behavior in five males over a cumulative 8 years. Fresh fecal samples, collected at GPS locations of collared individuals, were analyzed for FTM. In total, 111 feces were collected in 2022–2023 across 58 locations. The FTM values and the four behavioral variables were compared between the breeding (May–July) and post‐breeding season (August–November) using the Brunner–Munzel test. Overall, FTM were higher, while movement speed and activity levels were lower during the breeding season than in the post‐breeding season. These results suggest that some males may engage in consorting associated with elevated testosterone. We further examined the relationships between FTM and the four behavioral variables using Spearman's correlation tests. The correlation between FTM and behaviors varied among males. For example, the relationship between FTM and net movement distance was negative in two males but positive in three males. These interindividual differences in testosterone–behavior correlations were possibly the result of incomplete sampling during mating and consorting, as well as variation in breeding strategies. The negative relationships may indicate mate guarding or consorting, whereas positive relationships may reflect roaming. Our results suggested that the behavior of male Asian black bears may be influenced by increased testosterone, and monitoring testosterone allows us to gain insights into male bear behavior.

## Graphical abstract

Comparisons of mean fecal testosterone metabolite concentrations (mFTM) and behavioral variables between the breeding (May–July) and post‐breeding (August–November) seasons. The asterisks indicate significant differences.

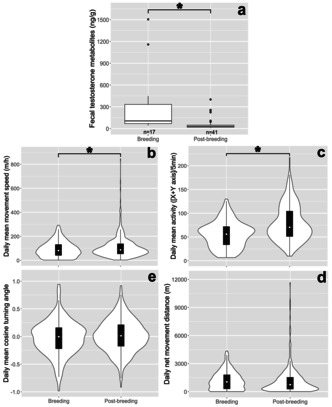

## 1 Introduction

Testosterone, a key sex hormone in male vertebrates, plays a critical role in breeding and reproductive behaviors, and is directly involved in spermatogenesis (Malo et al. 2009; Wingfield et al. 1990). During spermatogenesis, testosterone helps prevent cell death of spermatogenic cells, thereby supporting the production of viable sperm (Blottner et al. 1996; Tenniswood et al. 1992; Thompson 1994). Increasing gonadal testosterone secretion enhances reproductive behaviors that support success of mating and reproduction (Hau 2007). During the breeding season, males exhibit characteristic behaviors for mating with females, such as consorting, roaming, and aggression. Roaming behavior is characterized by the expansion of the home range (Clutton‐Brock 1989; Fisher and Lara 1999) and by increased movement distances or speeds (Edelman and Koprowski 2006; Falcinelli et al. 2024) in order to locate receptive females (Foley et al. 2015). When a male successfully encounters a potential mate, he exhibits consorting behavior—staying close to the female for several days, mating multiple times, and preventing copulation by rival males (Clutton‐Brock 1989; Rasmussen 1986; Stirling et al. 2016). Aggressive behavior also occurs during mate‐guarding, involving direct male–male competition for access to receptive females (Nie, Zhang, et al. 2012; Nie, Swaisgood, et al. 2012; Girard‐Buttoz et al. 2014). Notably, testosterone influences those aggressions, especially in species with a promiscuous mating system (Hirschenhauser and Oliveira 2006). Similarly, in male Giant panda ( *Ailuropoda melanoleuca* ), testosterone levels were elevated during encounters and consorting with estrous females (Nie, Zhang, et al. 2012). Overall, testosterone is widely recognized as a key hormonal driver of aggression and reproductive activity in vertebrates (Wingfield et al. 1990). These relationships between testosterone and reproductive behaviors have been confirmed through blood and fecal hormone measurements, as well as direct observation of behavior. While many testosterone‐related reproductive behaviors—such as aggression and mating—have been documented, the relationship between testosterone and movement patterns remains unclear. For example, a study on the large psammodromus (*Psammodromus algirus*) found that testosterone did not influence movement or home range size but did promote aggressive behavior (Salvador et al. 1996). However, comparable studies focusing on mammalian species—particularly large mammals—are lacking. In general, the relationships between reproductive behaviors and testosterone have been documented through behavioral observation for wild animals (i.e., Dittami and Reyer 1984; Nie, Zhang, et al. 2012; Nie, Swaisgood, et al. 2012; Preston et al. 2012; Salvador et al. 1996; Surbeck et al. 2012). Yet, this approach presents significant challenges when applied to studying behavioral patterns in elusive species, especially carnivores, due to their wide‐ranging activity and long movement distances, which makes continuous observation difficult (Naganuma et al. 2021; Sells et al. 2022).

The Asian black bears ( *Ursus thibetanus* ) is a large forest‐dwelling carnivore distributed across Asia. In reproduction, this species is a seasonal breeder with a promiscuous mating system (Tomiyasu et al. 2021; Yamamoto et al. 1998). In both captive and free‐ranging males, testosterone levels begin to rise during the pre‐breeding season (March–April) and peak during the breeding season (May–July) (Komatsu et al. 1997; Tomiyasu et al. 2021). In captive individuals, testosterone levels also increase both before and after mating (Chang et al. 2009). The other ursid species also had been conducted of monitoring testosterone, such as Brown bear ( *Ursus arctos* ) (Anel‐López et al. 2017; Bryan et al. 2014; Tsubota and Kanagawa 1989; White et al. 2005), Polar bear ( *Ursus maritimus* ) (Ciesielski et al. 2023; Howell‐Skalla et al. 2002; Palmer et al. 1988), American black bear ( *Ursus americanus* ) (Garshelis and Hellgren 1994; Mcmillin et al. 1976; Brito et al. 2011). In the wild, males exhibit extensive roaming behavior to locate receptive females during the breeding season (Baek et al. 2025). Once a male successfully approaches a female, the male engages in consorting behavior, staying with the female for several days to mate and displaying mate‐guarding behavior to deter rival males (Naganuma et al. 2021). Those reproductive behaviors were also confirmed in other bear species, such as roaming behavior in Brown bear (Dahle and Swenson 2003; Falcinelli et al. 2024; Steyaert et al. 2012). In consorting behavior, Polar bear had been observed that male and female remain together for several days or week until mating (Stirling et al. 2016). However, the relationship between seasonal changes in testosterone levels and reproductive behaviors or movement patterns in Asian black bears remains poorly understood. In particular, it is unclear whether both roaming and consorting which represent contrasting behavioral strategies, are influenced by increasing testosterone levels.

Here, we examined the relationship between testosterone levels and reproductive behavior in free‐ranging male Asian black bears through all seasons without hibernation, by combining fecal testosterone metabolite (FTM) analysis with GPS telemetry, activity sensor, and animal‐borne cameras. We tested two contrasting hypotheses: (1) when FTM concentrations increase, males exhibit roaming behavior for searching and encountering females; and (2) elevated FTM concentrations correspond with consorting behavior for staying near a female to ensure mating success.

## 2 Materials and Methods

### 2.1 Study Areas

**FIGURE 1:**
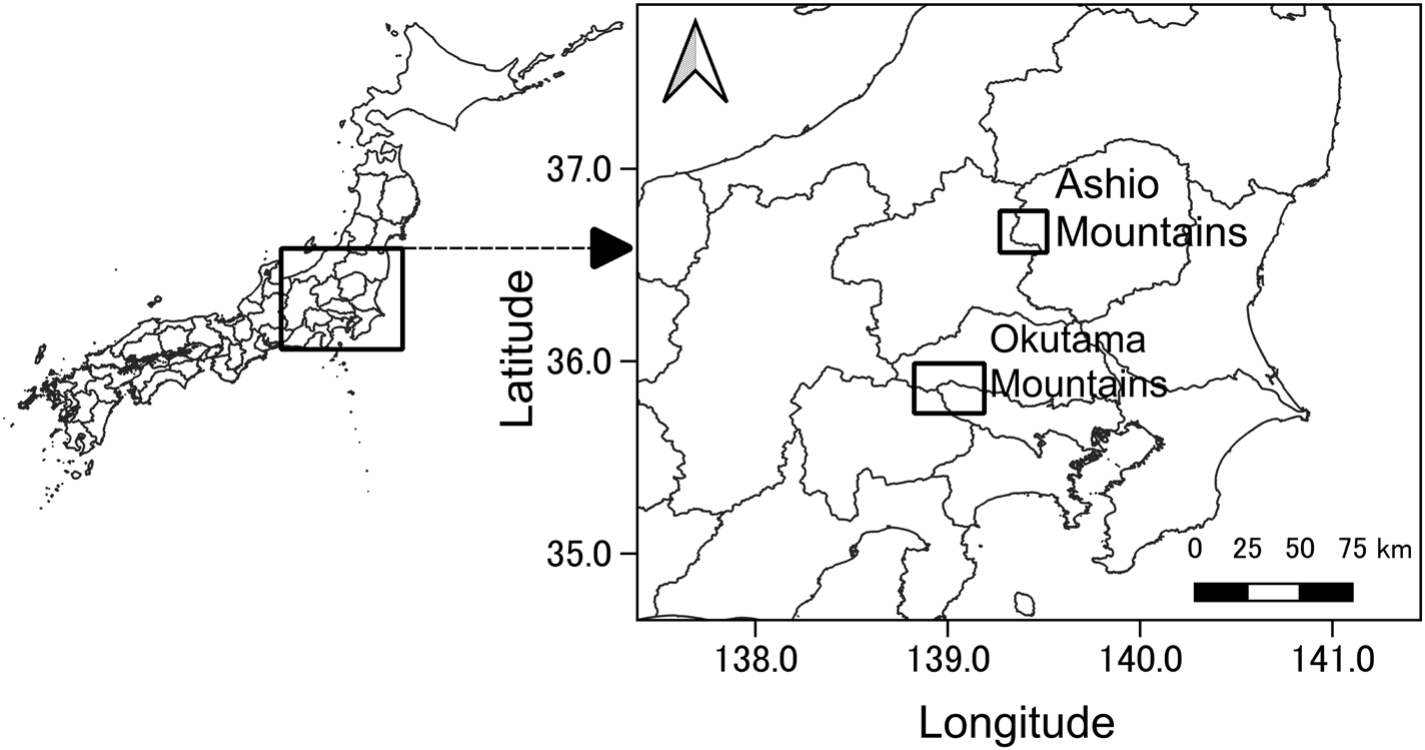
Study areas. Our study was conducted in two areas in central Honshu Island, Japan: The Ashio‐Nikko Mountains and the Okutama Mountains. Geographical and meteorological details of the areas are presented in “Study areas” in the main text. The two squares in the figure indicate the approximate area of each study area, but are not shown to scale.

The study was conducted in two areas in central Honshu, Japan: the Ashio‐Nikko Mountains and the Okutama Mountains (Figure 1). The Ashio‐Nikko Mountain area (Ashio Mountains) (36.44°–36.80° N, 139.22°–139.49° E) is steep terrain with an elevation ranging from 400 to 2400 m a.s.l. The annual precipitation and annual mean temperature were 1830.0 mm and 7.7°C in 2022, and 1909.0 mm and 8.8°C in 2023, as measured at the Oku‐Nikko Meteorological Station (36.7° N, 139.5° E, 1291.9 m a.s.l; Japan Meteorological Agency 2025). The Ashio Mountains are covered with broad‐leaved forest composed of Japanese oak (*Quercus crispula*), Jolcham oak ( *Q. serrata* ), maple (*Acer* spp.) and Japanese clethra (*Clethra barbinervis*) up to 1600 m a.s.l. Above 1600 m, mixed forests of Japanese hemlock (*Tsuga* spp.) and birch (*Betula* spp.) occur. Plantations of Japanese cedar ( *Cryptomeria japonica* ) and Japanese cypress ( *Chamaecyparis obtusa* ) are common below 1000 m a.s.l., and plantations of Japanese larch ( *Larix kaempferi* ) are common from 1000 to 1600 m a.s.l.

Okutama Mountains (35.79°–35.97° N, 138.77°–139.15° E) is also located on steep terrain with an elevation ranging from 400 to 2000 m a.s.l. The annual precipitation and annual mean temperature were 1286.0 mm and 12.5°C in 2022, and 1267.5 mm and 13.6°C in 2023, as measured at the Ogouchi Meteorological Station (35.79° N, 139.05° E, 530 m a.s.l.; Japan Meteorological Agency 2025). The Okutama Mountains is mostly covered with a mixed mosaic of forest vegetation (Koike et al. 2008). Conifer plantations cover 50% of the area and include Japanese cedar, Japanese cypress, and Japanese larch. Natural forest covers 40% of the area, which contains chestnut ( *Castanea crenata* ) and Jolcham oak from 400 to 500 m a.s.l.; Japanese oak, chestnut, and Japanese beech (*Fagus crenata*) from 500 to 1500 m a.s.l.; and conifer trees ( *Abies homolepis* and *Tsuga diversifolia*) from 1500 to 1800 m a.s.l.

### 2.2 Bear Captures and Behavioral Data Collection

We captured Asian black bears in the Ashio Mountains from 2021 to 2023 and in the Okutama Mountains from 2022 to 2023. Bears were captured by using handmade barrel traps as previously described in Takekoshi et al. (2024). Captured bears were immobilized using a combination of tiletamine hydrochloride and zolazepam hydrochloride (Zoletil, 8 mg/kg estimated body weight; Virbac, Carros, France). Sex was confirmed, and the upper first premolar was extracted for age determination via counting annual cementum layers as previously described (Tochigi et al. 2019). Blood samples were collected into heparinized Venoject II vacuum tubes (Terumo Inc., Tokyo, Japan) for DNA analysis. Standard body measurements, including body weight, were recorded.

From 2021 to 2023, we captured 25 bears (13 males and 12 females). For the purpose of this study, we used GPS location data for five males over a total of 8 years excluding hibernation period (December–April) (Table 1). One bear (AM97) was captured twice in 2021 and 2023, respectively.

**TABLE 1:** Summary of the five bears examined. Body weight was recorded on the captured site.

Bear ID	AM54	AM97	AM97	OM114	OM116	OM118
Study area	Ashio	Ashio	Ashio	Okutama	Okutama	Okutama
Years with GPS collar data	2022	2021–2022	2023	2022–2023	2022–2023	2023
Age (years)	13	5–6	7	8–9	7–8	3
Body weight (kg)	87	43	58	74	85	36
Data obtained from GPS collar	Location	Location activity	Location activity camera video	Location activity	Location activity	Location activity
Survey years	2022	2022	2023	2022–2023	2022–2023	2023
The number of fecal samples	13	21	12	34	13	18
Feces collection sites	7	11	7	16	9	8

For the bears, we equipped GPS collars (Vertex Plus; Vectronic Aerospace GmbH, Berlin, Germany) of three types: 2D battery model (810 g), 3D battery model (1200 g), and camera‐equipped model (1200 g) (Table 1). We dropped the collars using remote control at the end of collar battery. Those collars had been equipped from 5 months to 1.5 years in 2D battery model, 1.5 years in 3D battery model, and 2 months in camera‐equipped model. All capture and handling procedures followed the ethical guidelines of Mammal Society of Japan (https://www.mammalogy.jp/guideline2023_English.html). The collar weights for male bears did not exceed 3% of their body weight (Table 1).

The 2D and 3D battery collars were programmed to record GPS locations every 2 h. The camera‐equipped collars were programmed to record GPS locations and a 10‐s video clip every 30 min. Among the camera‐equipped collars, recording video clips was programmed as a duty cycle of 13 h on (5:00–17:30) and 11 h off, based on the sunset/sunrise time in June and July in the study area (Naganuma et al. 2021). GPS location data were transmitted via the Iridium satellite network, while activity and video data were stored onboard and retrieved only after collar retrieval.

The individuals varied in age (Table 1), including one sub‐adult (OM118); however, as male bears reach sexual maturity at 3 years old (Komatsu et al. 1994; Tomiyasu et al. 2021), and there were no significant differences in FTM levels among the five males through all seasons (Kruskal–Wallis test; *p* = 0.527; performed by R packages *PMCMRplus* (Pohlert 2024)). Therefore, we did not categorize them by age class or exclude the sub‐adult from the analysis.

Following the method of Kozakai et al. (2013), we excluded GPS location, activity, and video data for 7 days after immobilization for first‐time captured bears and for 3 days after immobilization for bears captured multiple times, allowing for acclimation to the collars. Additionally, the day of remote collar drop was excluded to avoid bias, as we approached the bears within the range of feasible remote control (approximately < 500 m). GPS locations with over 10 of the dilution of precision were also excluded to ensure accuracy of GPS location (D'Eon and Delparte 2005).

### 2.3 Fecal Sample Collection

We collected fecal samples from May to November 2022 and from May to October 2023. Samples were collected from clusters of GPS locations where GPS‐collared male bears had stayed for at least 2 h. We also collected feces when we found them in the field opportunistically. We approached these location clusters as soon as possible to obtain fresh feces. However, a delay of up to approximately 4 days (mean = 35, 1–86 h) occurred between the GPS fix and fecal collection because GPS location data were transmitted via the Iridium satellite network in near real‐time. To assess whether exposure time affected FTM concentrations, Takekoshi et al. (unpublished data) conducted an experiment using nine feces collected immediately after defecation from a captive male Asian black bear at a zoo. The results indicated that FTM concentrations of almost fecal samples did not change significantly between non‐exposure portions and exposed it through 1 to 96 h after defecation (Takekoshi et al., Unpublished manuscript). Therefore, we considered that the time elapsed between defecation and sample collection did not influence the FTM concentrations in this study.

To minimize contamination, fecal samples were collected using plastic bags turned inside out over the researcher's hand to avoid direct contact (Rosenbaum et al. 2021). Samples were taken only from the upper portion of boluses to retrieve fresher and cleaner sections while avoiding those that had come into contact with the ground (Hinchcliffe et al. 2021). Since steroid metabolites are not evenly distributed throughout the feces (Palme 2005), samples were well homogenized inside the plastic bags in the field. The samples were transported in a cooler bag with ice packs and stored at −20°C freezer in a laboratory or research station on the same day until hormone extraction.

### 2.4 DNA Analysis for Identification of Fecal Samples

DNA analysis was conducted to determine the individual to which each sample of feces belonged. To collect fecal DNA, we wiped the surface of the fecal samples carefully with a flocked swab (Flocked Swab 25‐3706‐H; Sugiyama‐gen Co. Ltd., Tokyo, Japan) and then placed the swab in a 2 mL tube containing InhibitEX Buffer (Qiagen Inc., Tokyo, Japan), according to Shimozuru et al. (2020). The swabs were stored at −20°C until DNA extraction using a Qiagen QIAamp Fast DNA Stool Mini kit (Qiagen Inc.). We also extracted DNA from blood samples collected in the capture site, using a MagExtractor kit (Toyobo, Osaka, Japan). Genotypes at 14 DNA microsatellite loci (G1A, G1D, G10B, G10J, G10L, G10P, G10X, MSUT‐1, MSUT‐2, MSUT‐6, MSUT‐7, UarMU05, UarMU23, and UarMU50; Kitahara et al. 2000; Paetkau et al. 1995, 1999) were determined by polymerase chain reaction in all samples, according to Takayama et al. (2023). Genotyping was performed twice to confirm the consistency of the results. To match the individuals to the fecal samples, we compared the genotypes of fecal samples with those of blood samples collected from GPS collared bears in CERVUS 3.0.7 (Kalinowski et al. 2007). CERVUS calculates two probabilities: pID, which is the probability that a single unrelated individual has the target genotype, and pID‐SIB, which is the probability that a single full sibling has the target genotype (Waits et al. 2001). We used these two probabilities as criteria to assess the accuracy of the matches between the bear and fecal genotype. Although pID‐SIB is designed to estimate the probability of siblings' having the same genotype (Waits et al. 2001), we used it here only as a more precise way to identify individuals than pID.

### 2.5 Hormone Analysis

Frozen feces were dried completely at 100°C overnight (> 12 h) on the electric muffle furnace, and pulverized using a pestle and mortar. Then, 0.1 g of the powder was placed in a centrifuge tube containing 5 mL of 80% methanol. The tube was shaken for 30 min by using a vortex mixer and then centrifuged at 1645 *g* for 10 min, and 2 mL of the supernatant containing the feces extract was moved to a new centrifuge tube and stored at −30°C until assay.

Fecal testosterone was measured by using a competitive double‐antibody enzyme immunoassay, as described in a previous study (Yanagawa et al. 2015). Twenty microliters of samples were incubated for 16–18 h at 4°C with the primary antisera and horseradish peroxidase (HRP)‐labeled hormone (100 μL each) in the well of a 96‐well microplate (Costar 3590, Corning, NY, USA) coated with the secondary antiserum. Afterall the wells were washed for 4 times with 300 μL of washing buffer (0.05% Tween 80), 150 μL of Tetramethylbenzidine (TMB) solution (5 mM citric acid, 50 mM Na_2_HPO_4_, 500 mM Urea hydrogen peroxide (UHP), 1 mM TMB and 2% Dimethyl sulfoxide (DMSO)) was added to each well, and the mixture was incubated for 40 min at 37°C. After the chromogenic reaction was stopped with 50 μL of 4 N H_2_SO_4_, the absorbance of the solution in the wells was read by a microplate reader (iMark, Bio‐Rad Laboratories, Tokyo, Japan) at 450 nm. The primary antisera used for the assay of testosterone was anti‐testosterone‐3‐BSA (MBS534956, MyBioSource, CA, USA) which cross‐reacted with androstenedione (0.5%) and Dehydroepiandrosterone (DHEA) (0.01%). Goat anti‐rabbit serum (111‐005‐003, Jackson Immuno Research, PA, USA) was used as the secondary antiserum. All samples were assayed in triplicate. The assay sensitivities were 0.4 pg/well. The intra‐ and inter‐assay coefficients of variation were 4.3% and 8.4%, respectively.

### 2.6 Assumption of Defecation Date and Calculation of Mean FTM

The collection of newer fresh feces was prioritized to try to reduce the impact of exposure to bacterial enzymes on testosterone levels in older fecal samples (Palme 2005). Based on Sergiel et al. (2020), we evaluated freshness of the feces based on smell and appearance (wetness, color, and degree of decomposition). However, in this study, the average time that bears stayed at locations where we collected feces was over a day (mean = 26; 2–234 h) based on GPS location. Even though the actual defecation date could not be detected, we assigned that the defecation date was the date of the last fixed GPS location at the cluster where feces were collected, and the collected fresh feces were assumed to have been defecated within the assigned defecation date. In several locations where fecal samples were collected, we gained multiple fecal samples. Despite being collected from the same location, FTM concentrations varied between individual samples. To account for this variability, we calculated the mean FTM (mFTM) values for each location where fecal samples were collected.

### 2.7 Calculating Behavioral Variables

To analyze movement patterns, we first resampled the GPS location data of all collars, including a GPS camera collar scheduled at a 30 min fixed interval, at a fixed interval of 2 h. We then calculated movement distance and turning angles using the ‘prepData’ function in the R package *moveHMM* (Michelot et al. 2016). From these calculations, we derived three behavioral variables: (1) daily mean movement speed—the mean hourly movement distance per day, divided by the time interval (2 h) between consecutive GPS locations; (2) daily net movement distance—the straight‐line distance between the initial and final locations within a day; and (3) daily mean cosine turning angle—the cosine of the turning angle calculated using three consecutive GPS locations.

In addition to GPS data, we used the dual‐axis motion sensors in the collars to assess the bears' activity levels. These sensors continuously record head and neck movements along the side‐to‐side (*x*‐axis) and up‐down (*y*‐axis) directions at 5 min intervals, with a maximum count of 255 per axis. That motion sensor reflects whole body moves (Kozakai et al. 2008). Using these motion sensor data, we calculated one final variable: (4) daily mean activity—the daily mean value of total *x*‐axis and *y*‐axis counts per 5 min interval, calculated as ([*x*‐axis count + *y*‐axis count]/5 min). All data processing and calculations were performed in R (v. 4.4.2; R Core Team 2024).

### 2.8 Statistical Analyses

To assess differences in testosterone levels and behaviors at the seasonal scale, we compared the mFTM values and those of the four behavioral variables between the breeding and post‐breeding seasons by using the Brunner–Munzel test, implemented via the ‘brunner.munzel.test’ function in the R package *lawstat* (Gastwirth et al. 2023). However, months with no fecal sample were excluded, as we could not validate whether behavior during those months was involved with testosterone. The relative effect (RE) of the Brunner–Munzel test, ranging from 0 to 1, indicates the probability of one group's values being higher or lower than another group's value (Breathett et al. 2023). The RE > 0.5 indicates that values in group X_1_ are lower than those in group X_2_, while the RE < 0.5 indicates that values in group X_1_ are higher than those in group X_2_ (Karch 2023). In this study, X_1_ represents the breeding season, and X_2_ represents the post‐breeding season for each variable. On the basis of behavioral studies of captive (Yamamoto et al. 1998) and free‐ranging (Naganuma et al. 2021) Asian black bears, as well as plasma testosterone data from free‐ranging individuals (Tomiyasu et al. 2021), we defined the breeding season as 1 May–31 July, and the post‐breeding season as 1 August–30 November.

To examine the relationship between testosterone and behavior, we calculated Spearman's correlation coefficients. Since fecal steroids reflect hormone secretion at a certain time before (Palme 2005), we matched behavioral data to the estimated timeframe when testosterone was likely secreted. The time delay from steroid secretion to defecation was roughly calculated by gut passage time (Palme 2005). On the basis of gut passage time in Asian black bears (Koike et al. 2011), which ranges from 3 to 44 h, steroid hormones—including testosterone—could have been secreted any time between 2 days before defecation and the day of defecation. Therefore, for each mFTM value, we subsampled behavioral variables from 2 days before defecation (2BD), 1 day before defecation (1BD), and the day of defecation (DD). We then tested the correlation between mFTM and each behavioral variable at 2BD, 1BD, and DD using the ‘cor.test’ function in the R package *stats* (R Core Team 2024). In addition, we estimated 95% confidence intervals for the Spearman's correlation coefficients using 1000 bootstrap replicates, implemented via the ‘spearman.ci’ function in the R package *RVAideMemoire* (Herve 2023). All statistical analyses were conducted in R v. 4.4.2.

### 2.9 Video Data Analysis From a Bear Equipped With a GPS/Camera Collar

From the video data of a GPS camera collar, we extracted the clips classified as mating behavior. To confirm whether testosterone level increases due to mating or not, we compared the mFTM concentration and the existence of mating at 2BD, 1BD, and DD. However, recording video time in the GPS camera collar was scheduled during daytime, as mentioned above, and we could not confirm whether reproductive behavior occurred during nighttime.

## 3 Results

We collected a total of 111 fecal samples in 2022 (May–November) and 2023 (May–October) and identified through DNA analysis as belonging to the five target bears (Table 1). Those samples were collected at 58 locations, of which 17 were during the breeding season and 41 during the non‐breeding season. Consequently, the analysis included 58 mFTM concentrations with corresponding behavioral variables at 2BD, 1BD, and DD.

**FIGURE 2:**
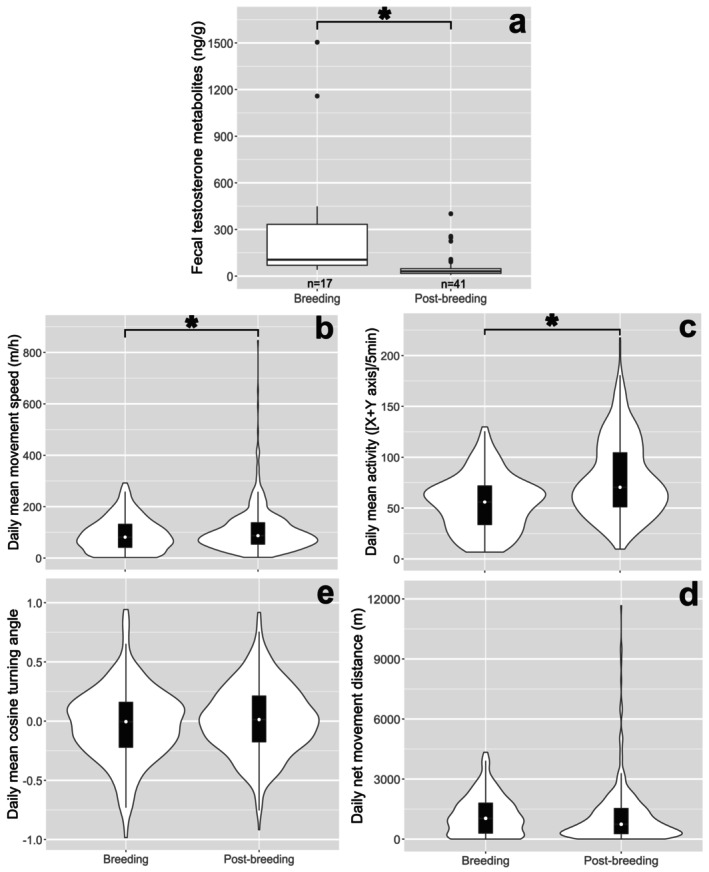
Comparisons of fecal testosterone metabolite concentrations and each of the indicated behavioral variables between the breeding (May–July) and post‐breeding (August–November) seasons. The asterisks (*) indicate significant differences between the breeding and post‐breeding season (Brunner–Munzel test; *p* < 0.05). The “*n*” under the boxplots in (a) shows the numbers of fecal samples. The width of white layers display density, white point on the black boxplot displays median value in (b–e).

mFTM concentrations and each of the behavioral variables were compared between the breeding and post‐breeding seasons (Figure 2). The mFTM concentration was higher during the breeding season than during the post‐breeding season (RE = 0.129, *p* < 0.001; Figure 2a). Daily mean movement speed and daily mean activity were both lower during the breeding season than during the post‐breeding season (mean movement speed: RE = 0.547, *p* = 0.031; mean activity: RE = 0.678, *p* < 0.001; Figure 2b,c). In contrast, daily net movement distance (RE = 0.459, *p* = 0.099; Figure 2d) and daily mean cosine turning angle (RE = 0.535, *p* = 0.097; Figure 2e) were comparable between the seasons.

**FIGURE 3:**
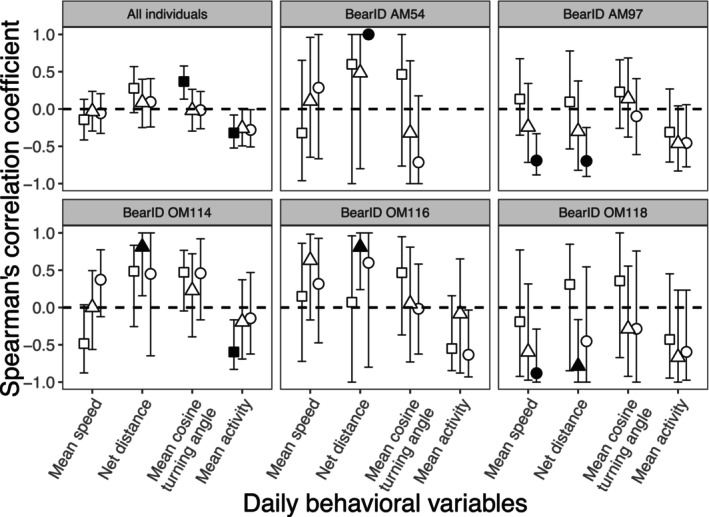
Spearman's correlation coefficients between mean fecal testosterone metabolite (mFTM) concentration and each of the daily‐scale behavioral variables of the five bears individually and combined. Coefficients: Squares represent 2 days before defecation (2BD), triangles represent 1 day before defecation (1BD), and circles represent the day of defecation (DD). The closed symbols of squares, triangles, and circles represent significant correlations between mFTM concentration and behavior. The dotted lines show zero of the Spearman's correlation coefficient. Error bars show 95% confidence intervals.

When the five bears were considered as a group, the correlation between mFTM concentration and daily mean cosine turning angle on 2BD was positive (*ρ* = 0.369, *p* = 0.004), and that between mFTM concentration and daily mean activity on 2BD was negative (*ρ* = −0.320, *p* = 0.022) (Figure 3). However, when bears were considered individually, several interindividual differences were observed (Figure 3). The correlation between mFTM concentration and daily mean activity on 2BD was negative only in OM114 (*ρ* = −0.596, *p* = 0.015). The correlation between mFTM concentration and daily mean movement speed on DD was negative only in bears AM97 (*ρ* = −0.690, *p* = 0.002) and OM118 (*ρ* = −0.881, *p* = 0.007). The correlation between mFTM concentration and daily net movement distance was negative on DD in AM97 (ρ = −0.697, *p* = 0.012) and on 1BD in OM118 (*ρ* = −0.786, *p* = 0.028), but significantly positive on DD in AM54 (*ρ* = 1.000, *p* = 0.003) and on 1BD in OM114 (*ρ* = 0.812, *p* = 0.008) and OM116 (*ρ* = 0.810, *p* = 0.022).

In 2023, seven feces were detected as AM97 using DNA analysis, which was tagged with a GPS camera collar. While those samples, mating was confirmed on the DD of only one feces, which is higher mFTM (defecated on 29‐July; 266.1 ng/g) than that of other feces (6‐August–2‐September; 21.2–256.2 ng/g) However, the former feces were defecated on breeding season and the latter feces were defecated during the post‐breeding season. Thus, the seven fecal samples could not be compared with the mFTM because of the different breeding seasons and the very limited sample size.

## 4 Discussion

Here, we examined the seasonal changes in testosterone and behavior in male Asian black bears. We found that increased FTM concentration corresponds with reduced movement, shorter distance, and more sinuous movement paths, supporting our second hypothesis.

### 4.1 Seasonal Differences in mFTM and Behavior

mFTM concentration was higher in the breeding season than in the post‐breeding season. This finding is consistent with previous findings on plasma testosterone (Tomiyasu et al. 2021) and testis size (Okano et al. 2003) in free‐ranging bears, as well as FTM concentration (Chang et al. 2009) and spermatogenic activity (Komatsu et al. 1997) in captive individuals.

Both daily mean movement speed and daily mean activity were lower in the breeding season than in the post‐breeding season. These seasonal differences suggest that reduced activity during the breeding season may be linked to mating behaviors such as mate guarding and consorting with females. Similar behaviors have been observed in male giant pandas, which stay at mating sites for several days to consort and guard mates (Nie, Zhang, et al. 2012; Nie, Swaisgood, et al. 2012). Likewise, mate guarding and consorting have been documented in brown bears (Spady et al. 2007; Steyaert et al. 2012) and Asian black bears (Naganuma et al. 2021). Therefore, some of the individuals in our study may have been engaging in mate guarding or consorting, with associated increased testosterone levels and reduced movement.

### 4.2 Correlation Between FTM Concentration and Behavior

Across all individuals, mean daily activity was negatively correlated with mFTM, and turning angles became more sinuous with increasing mFTM concentration; however, we found no relationship between mFTM concentration and daily mean movement speed or daily net movement distance.

At the individual level, two males showed a negative correlation of mFTM concentration with daily movement speed and daily net movement distance, while three males showed a positive correlation of mFTM concentration and daily net movement distance. This inconsistency suggests the use of different breeding behavioral strategies among individuals. The negative relationships between movement and testosterone secretion may indicate mate guarding or consorting (Nie, Zhang, et al. 2012; Steyaert et al. 2012), where males reduce movement while staying near a female. The positive relationships between movement and testosterone secretion may reflect roaming behavior, in which males increase movement to search for mates (Baek et al. 2025; Edwards and Derocher 2015; Falcinelli et al. 2024).

Male bears move farther during the breeding season than during the post‐breeding season (Falcinelli et al. 2024). Here, daily net movement distance was slightly longer during the breeding season, although the difference was not statistically significant. This variability may indicate that searching for females and mating‐related movement occurs repeatedly with a short period between each movement.

For example, in giant pandas, testosterone levels increase only when males are actively consorting with a female (Nie, Swaisgood, et al. 2012). Similarly, mountain gorillas show elevated fecal androgen metabolites immediately after mating (Rosenbaum et al. 2021). If Asian black bears also experience short‐term testosterone spikes during consorting and mating, our fecal sampling approach may have failed to capture these fluctuations. Thus, the limited duration of these events could explain why we did not find a consistent relationship between testosterone and behavior.

In summary, the behavior of male Asian black bears may be influenced by increased testosterone secretion, particularly during the breeding season due to higher testosterone levels. Thus, monitoring of sexual hormones offers a potentially valuable means of gaining insights into male bear behavior in the breeding season.

### 4.3 Relationships Between mFTM Concentration and Mating Behavior

Our study did not detect a direct association between testosterone level and observed copulation by animal‐borne camera. In red deer ( *Cervus elaphus* ), testosterone is tightly linked to sperm production (Malo et al. 2009) and appears to regulate copulation rate due to sperm availability, as shown for Soay sheep (Preston et al. 2012). By contrast, mating success in captive Asian black bear cannot be explained by testosterone level alone (Chang et al. 2009). Our study recorded slightly high mFTM on the day of mating in the free‐ranging Asian black bears, but our sample size was too small to clarify the tendency. Future studies are needed to collect larger FTM datasets and equip more males with animal‐borne cameras to clarify the relationship between testosterone secretion and mating behavior.

### 4.4 Limitation

The study has some limitations, particularly in sample size. Although fecal samples were collected every 1–2 weeks when the bears were active, we observed gaps of up to 2 months for some bears during the breeding season. During this period, food resources are generally scarce (Koike 2025), and male bears often reduce foraging while focusing on courtship behaviors (Naganuma et al. 2021). This may have decreased the defecation frequency during the breeding season, as the bears' behavior and food intake are likely influenced by these seasonal changes. In addition, our dataset was limited to only five bears and a total of only eight cumulative years, and some bears were fitted with GPS collars in the middle or later part of the breeding season (i.e., in June or July). Consequently, the available data on GPS locations, activity, and mFTM concentrations did not cover the full breeding and post‐breeding seasons. Future studies with a larger sample size and data covering the entire breeding season (May–July) are needed to more clearly define the relationship between testosterone secretion and behavioral patterns in male bears.

## Author Contributions

**Naoki Takekoshi:** conceptualization (lead), data curation (equal), formal analysis (equal), funding acquisition (equal), investigation (equal), methodology (equal), validation (equal), visualization (equal), writing – original draft (equal), writing – review and editing (equal). **Jumpei Tomiyasu:** conceptualization (lead), methodology (lead), project administration (equal), supervision (lead), writing – review and editing (equal). **Yojiro Yanagawa:** data curation (lead), formal analysis (lead), methodology (lead), resources (equal), writing – review and editing (equal). **Naoki Ohnishi:** data curation (lead), formal analysis (lead), methodology (equal), resources (equal), validation (equal), visualization (equal), writing – review and editing (equal). **Tomoko Naganuma:** data curation (lead), formal analysis (lead), funding acquisition (equal), methodology (lead), writing – review and editing (equal). **Seung‐Yun Baek:** data curation (equal), formal analysis (equal), investigation (equal), methodology (equal), software (equal), writing – review and editing (equal). **Miu Itoh:** investigation (supporting), writing – review and editing (supporting). **Xin Luo:** investigation (supporting), writing – review and editing (supporting). **Tatsuhito Nishiwaki:** investigation (supporting), writing – review and editing (supporting). **Chinatsu Kozakai:** data curation (lead), resources (equal), writing – review and editing (equal). **Shinsuke Koike:** project administration (lead), resources (equal), supervision (equal), writing – review and editing (equal). **Koji Yamazaki:** conceptualization (lead), funding acquisition (lead), investigation (equal), methodology (equal), project administration (lead), resources (equal), supervision (lead), validation (equal), writing – review and editing (equal).

## Funding

This work was supported by Doctoral Research Grant Program of Tokyo University of Agriculture, 46407378G; Sumitomo Foundation; Support for Pioneering Research Initiated by the Next Generation, JPMJSP2122; Japan Society for the Promotion of Science, 23K23654, 22H02389.

## Conflicts of Interest

The authors declare no conflicts of interest.

## Data Availability

The data of FTM concentrations and behavioral variables were available in Dryad at link: https://doi.org/10.5061/dryad.pg4f4qs2k.
